# A stratified therapeutic model incorporated with studies on regulatory B cells for elderly patients with newly diagnosed multiple myeloma

**DOI:** 10.1002/cam4.5228

**Published:** 2022-09-20

**Authors:** Wenjiao Tang, Yan Li, Zhongqing Zou, Jian Cui, Fangfang Wang, Yuhuan Zheng, Li Hou, Ling Pan, Bing Xiang, Hong Chang, Li Zhang, Ting Niu

**Affiliations:** ^1^ Department of Hematology Institute of Hematology, West China Hospital, Sichuan University Chengdu Sichuan China; ^2^ Department of Hematology Clinical Medical College & Affiliated Hospital of Chengdu University, Chengdu University Chengdu Sichuan China

**Keywords:** elderly, multiple myeloma, regulatory B cell, stratification, survival

## Abstract

**Objective:**

Despite the availability of new agents, elderly patients with multiple myeloma (MM) usually present with poor outcomes due to the heterogeneity of disease conditions, especially immune deficiency. Regulatory B cells (Bregs) can be involved in immune defects by exerting immune regulatory functions in MM. In order to provide more evidence‐based practice for the elderly MM, the study established and assessed a stratified therapeutic model with studies on Bregs for Chinese Elderly Multiple Myeloma in 2021 (CEMM2021).

**Methods:**

In this open‐label, non‐interventional, prospective study in the real world, 159 newly diagnosed MM (NDMM) patients over *65 years old* were sequentially recruited and bone marrow aspirates prior to treatment were obtained to detect the ratios of Bregs by flow cytometry.

**Results:**

Based on the CEMM2021 model, 147 patients had received at least one cycle of induction therapy, including bortezomib/dexamethasone (Bd) (*n* = 80), lenalidomide/dexamethasone (Rd) (*n* = 27), Bd with a third agent X (Bd + X) (*n* = 27), and other regimens (*n* = 13). The proportions of patients achieving very good partial response or better were comparable among Bd, Bd + X, and Rd groups (41.9% vs. 54.5% vs. 44.0%, *p* = 0.472). Besides, the progression‐free survival (PFS) and overall survival (OS) were not significantly different among Rd, Bd, and Bd + X groups. Multivariable analysis showed that induction efficacy less than partial response (PR) were poor prognostic factors for PFS, while Revised‐International Staging System (R‐ISS) III and efficacy less than PR were poor prognostic factors for OS. This study also found that the ratios of bone marrow Bregs <10% (*p* = 0.036) and SUVmax of PET‐CT scan >4.2 (*p* = 0.000) were closely correlated with OS in the elderly MM.

**Conclusions:**

For the elderly NDMM, the CEMM2021 algorithm in our center might provide a valuable reference for the guidance of therapeutic strategies, with the combination of Bregs resulting in an effective and clinically meaningful prediction in contemporary treatment.

## Graphical abstract

The management of elderly patients with MM remains challenging due to the heterogeneity of disease conditions, especially immune deficiency. In order to provide more evidence‐based practice for the elderly MM, the study established and assessed a stratified therapeutic model with studies on Regulatory B cells for Chinese Elderly Multiple Myeloma in 2021 (CEMM2021).

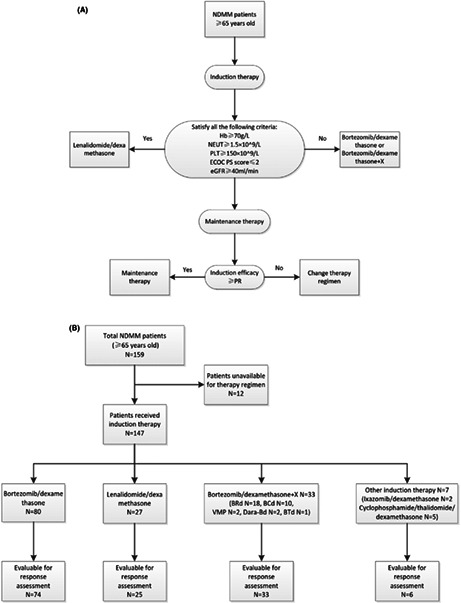

## 1 INTRODUCTION

Multiple myeloma (MM), which is the second most common hematological tumor with the mean annual age‐adjusted incidence of around six per 100,000 per year in the European and American areas,^1^ occurs mainly in the elderly, with the median age at diagnosis being approximately 70 years old in European and American areas^2^ and 58 years old in Asian areas.^3^ The accumulation of elderly patients with MM has definitely emerged as considerable attention to the challenge of *global aging*. Although the availability of new agents including bortezomib and lenalidomide dating from the *beginning of the* 21st *century*, elderly MM patients usually present poor outcomes due to the heterogeneity of disease conditions,^4^ resulting in shorter survival compared with younger patients.^5^, ^6^ In general routine practice, *elderly patients are often frail* with multiple comorbidities and immune deficiency,^7^ contributing to their intolerance of intensive chemotherapy or autologous stem cell transplantation and *their tendency* to be excluded from randomized clinical trials. Therefore, it is essential to develop a stratification system to tailor the therapeutic approach for elderly MM patients across each region in the real world.^4^ Recently, the International Myeloma Working Group (IMWG) has proposed a scoring system to grade the frailty of elderly patients according to comorbidities, age, and mental and physical conditions to predict survival and risk of toxicity from treatment in elderly myeloma patients.^8^ However, it evaluated the patients enrolled in clinical trials, and it still lacks a study regarding the stratified therapeutic model for the elderly MM in real‐world practice, especially focusing on the role of the tumor‐infiltrating immune background.

Recently, regulatory B cells (Bregs) have been proven to be involved in immune defects by exerting immune regulatory functions.^9^ B cells have initially been found to mediate humoral immune responses through producing antibodies and also perform antigen‐presenting functions to promote T‐cell activation and proliferation.^10^ However, emerging studies have recently identified a small subset of B cells, namely Bregs, both in mice and humans, which can maintain immune tolerance and suppress responses during cancer immune surveillance by producing immune‐suppressive cytokines such as IL‐10 or IL‐35.^11^, ^12^, ^13^ It is noteworthy that recent studies have established genetic models to explore the potential of Bregs for therapeutic intervention in the setting of solid cancers.^14^ However, the research on Bregs for MM *up to now* is limited. Our previous studies have identified that the bone marrow (BM) derived Bregs presented with phenotype CD19^+^CD24^hi^CD38^hi^ conferring an immunosuppressive BM microenvironment in MM and their proportions within CD19^+^ B cells were correlated with treatment efficacy and prognosis.^15^ However, due to the elderly MM displaying *mark phenotypic heterogeneity*, the role of Bregs in predicting baseline immune condition and the clinical prognosis has not been illuminated.

Aiming to provide more evidence‐based practice for elderly patients with MM, the study established and assessed a stratified therapeutic model with studies on Bregs for Chinese Elderly Multiple Myeloma in 2021 (CEMM2021). First, the CEMM2021 model designed personal total therapy plans for the elder newly diagnosed MM (NDMM) at a referral *center* in *Southwest China*, based on *individual* clinical categories, such as predictions of adverse drug effects according to baseline testing, including complete *blood count and biochemistry profiles, as well as individual* genetic susceptibility, medical *comorbidities*, secondary *conditions*, and other health issues. Furthermore, the impact of Bregs has been evaluated to identify the associated aberrant immunophenotypic changes in elder NDMM. Finally, we have illustrated that the approach combining the CEMM2021 model with Bregs would be applicable for elder NDMM in the real world.

## 2 METHODS

### 2.1 Patients and study design

The prospective study was designed to be an open‐label, noninterventional, observational study in real‐world clinical practice, detailing how to optimize and stratify the use of current therapy regimens for elderly patients with NDMM (chiCTR2000029925). NDMM patients 65 years old and above were sequentially recruited between January 1, 2017 and April 1, 2021 and followed until July 10, 2021. This study was approved by the Ethics Committee of West China Hospital, Sichuan University (WCHSCU). All subjects provided written informed consent.

The inclusion criteria were previously untreated and symptomatic MM patients 65 years of age or older and ineligible for hematopoietic stem cell transplantation. The exclusion criteria included patients participating in other clinical trials. In addition, detailed baseline clinical data were collected, including Eastern Cooperative Oncology Group (ECOG) performance status, disease stage, and laboratory test results. The definition of the high cytogenetic risk group was in accordance with the IMWG definition,^16^ with the presence of *t* (14;16), *t* (4;14), and deletion (17p) detected by fluorescent in situ hybridization (FISH) of the CD138‐enriched plasma cells from the BM. The therapy efficacy was assessed based on the response criteria by IMWG.^17^ The best response referred to the maximum response achieved at any time during the initial therapy. The overall response rate (ORR) was equal to the partial response (PR) rate and better. Overall survival (OS) was defined as the duration from diagnosis to death or the last follow‐up. Progression‐free survival (PFS) was measured from diagnosis to disease progression, death, or the last follow‐up.

### 2.2 Treatment algorithm

**FIGURE 1:**
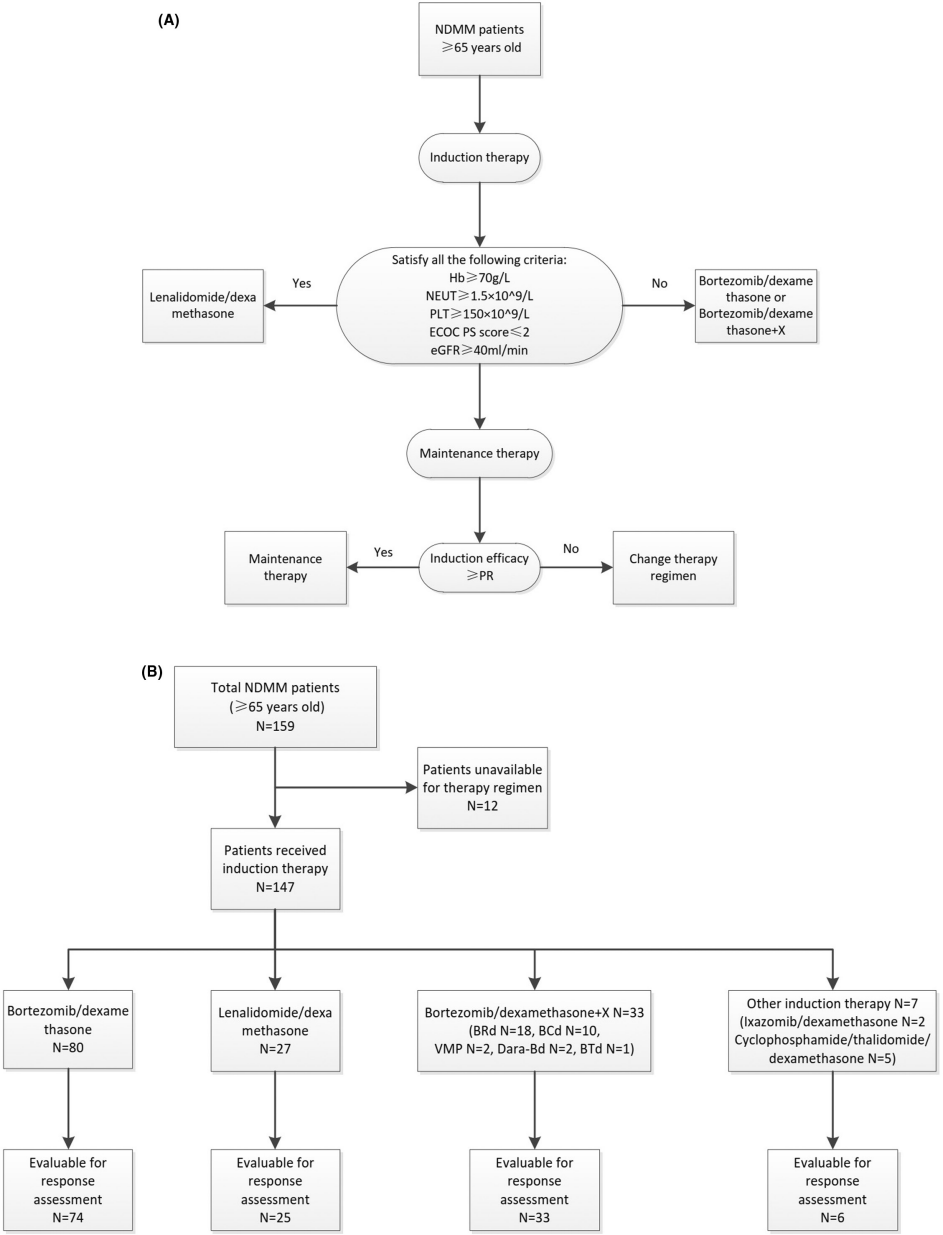
The treatment algorithm and details for the elderly newly diagnosed with multiple myeloma. BCd, bortezomib/cyclophosphamide/dexamethasone; BRd, bortezomib/lenalidomide/dexamethasone; BTd, bortezomib/thalidomide/dexamethasone; Dara‐Bd, daratumumab/bortezomib/dexamethasone; VMP, bortezomib/melphalan/prednisone.

To ascertain the best possible qualitative stratification, the final reasonable treatment decisions for all the enrolled patients were discussed with the *patient* and families according to our algorithm (Figure 1). The selection of induction therapy was mainly based on the following stratification standard. The inclusion criteria of *lenalidomide* plus low‐dose dexamethasone (Rd) regimen, which was demonstrated by the phase 3 *FIRST trial as the best option for contemporary elder patients with NDMM* ^*19*^, include hemoglobin ≥70 g/L, neutrophile granulocytes ≥1.5 × 10^9^/L, platelets ≥150 × 10^9^/L, creatinine clearance ≥40 ml/min/m^2^, and ECOG ≤ 2 points. The others receiving bortezomib‐based regimens would be adjusted according to individual health status, comorbidities, and risk stratification. Maintenance therapy was offered to patients achieving at least a PR as defined by the IMWG response criteria. Patients whose best response did not achieve PR would be transferred to another induction regimen. Specifically, the induction therapies were categorized as lenalidomide/dexamethasone (Rd), bortezomib/dexamethasone (Bd), and Bd + X regimens. Rd was given as lenalidomide 25 mg daily on days 1–21 of each 28‐day cycle and dexamethasone 10 mg/m^2^ on days 1–2, 8–9, 15–16, and 22–23. Bd was given as bortezomib subcutaneously 1.3 mg/m^2^ weekly, along with dexamethasone at a dose of 10 mg/m^2^ given separately on days 1–2, 8–9, 15–16, and 22–23 in a 4‐week cycle. Bd + X indicates that a third agent termed X, including cyclophosphamide, pegylated liposomal doxorubicin, or lenalidomide, was added to Bd. Specifically, for MM patients accompanied by renal impairment and/or amyloidosis, cyclophosphamide was administered with Bd regimens at a dose of 300 mg/m^2^ weekly. Meanwhile, MM patients with extramedullary disease would accept Bd along with pegylated liposomal doxorubicin at a dose of 40 mg for each cycle. Dose adjustments were made based on the physicians' discretion and patients' tolerability.

### 2.3 Examination of bone marrow‐derived CD19^+^CD24^hi^CD38^hi^ Bregs

The detailed procedures of examination of BM‐derived Bregs by flow cytometry were described in our previous work.^15^ Briefly, the BM aspirates of the elderly NDMM patients were obtained prior to the initial therapy, and mononuclear cells were isolated by gradient centrifugation on Ficoll for 20 min at 800 *g*, followed by transferring the lymphocyte layer into the serum‐free RPMI 1640 medium and centrifugation for 7 min at 2000 rpm. After discarding the supernatant, 1 ml of red blood cell (RBC) lysis buffer was added to lyse erythrocytes for 3–5 min, followed by centrifugation for 5 min at 2000 rpm. The remaining cells were washed with phosphate‐buffered saline (PBS) and then incubated with fluorescence‐labeled antibodies, including fluorescein isothiocyanate (FITC) antihuman CD19 antibodies, PE/Cy7 antihuman CD38 antibodies, and allophycocyanin (APC) antihuman CD24 antibodies (BioLegend, San Diego, CA) for 15 min. Finally, the antibody‐bound cells were resuspended in PBS for the six‐color flow cytometry detection (Beckman) after the removal of the unbound antibodies.

### 2.4 FDG‐PET/CT scan

The operation of FDG‐PET/CT scan was carried out following the guidelines of the European Association of Nuclear Medicine.^18^ In order to standardize the analysis of the PET/CT scan results, we adopted the criteria from the works reported by Bologna and Udine, which were briefly described in our previous work.^19^ In this study, SUVmax >4.2 was adopted as a demarcation indicator for unfavorable survival, which has also been endorsed in previous studies.^20^, ^21^, ^22^

### 2.5 Statistics analysis

The analysis of differences between groups was calculated by the t test or ANOVA for continuous variables and the chi‐square test or Fisher's exact test for categorical variables. Univariate analysis of survival curves was conducted according to the Kaplan–Meier method and log‐rank test. Cox proportional hazards model was applied for multivariate analysis of the prognostic factor for survival. A two‐sided α error of 0.05 was considered the statistical significance threshold. All the statistical analyses were performed by the SPSS 25.0 software and plotted by GraphPad Prism 8.0.

## 3 RESULTS

### 3.1 Baseline characteristics

**FIGURE 2:**
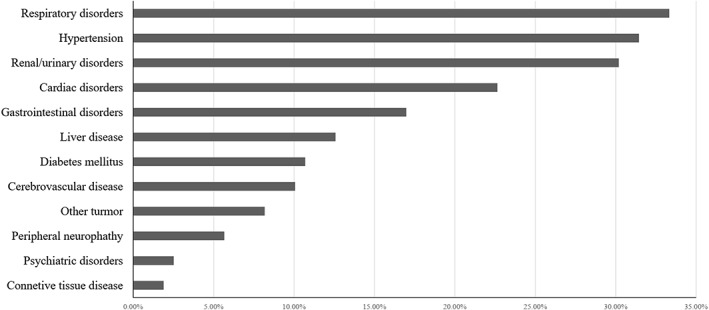
The ratios of comorbidities in the elderly multiple myeloma patients at enrollment.

Between January 1, 2017 and April 1, 2021, a total of 159 NDMM no younger than 65 years old were recruited with a median age of 70 years old and 25.2% of MM patients were more than 75 years old. The baseline clinical characteristics of the elderly MM patients were shown in Table 1. More than half of the patients had ECOG PS scores ≥2 and 28.9% presented with renal insufficiency. In addition, cytogenetic data were available for 105 elderly MM patients, of which 44.8% showed positive for 1q21 amplification. The percentage of patients with ISS III was higher than R‐ISS III (48.0% vs. 25.8%), although 34.0% presented with elevated lactate dehydrogenase (LDH) and 19.0% were classified as high risk according to the IMWG definition. The comorbidities of the elderly MM at enrollment were summarized in Figure 2 and the most common organ involved was the respiratory system (33.3%), such as pneumonia, chronic obstructive pulmonary disease, respiratory failure, and obstructive sleep apnea‐hypopnea syndrome. Other common comorbidities included hypertension, renal/urinary disorders, and cardiac disorders. Thirteen patients presented with a second tumor at enrollment like lung cancer, liver cancer, breast cancer, and other malignancy.

**TABLE 1:** Baseline characteristics and treatment efficacy of the elderly multiple myeloma patients

Characteristics– no (%)	Overall (*n* = 159)	Bd (*n* = 80)	Bd + X (*n* = 33)	Rd (*n* = 27)	*p* value^‡^
Median age (range)	70(65–88)	69(65–88)	69(65–81)	72(65–85)	**0.001**
65–74 years	119(74.8)	62(77.5)	31(93.9)	14(51.9)	
≥75 years	40(25.2)	18(22.5)	2(6.1)	13(48.1)	
Gender					0.329
Male	92(57.9)	48(60.0)	20(60.6)	14(51.9)	
Female	67(42.1)	32(40.0)	13(39.4)	13(48.1)	
Hemoglobin					0.642
<70 g/L	29(18.2)	17(21.3)	5(15.2)	4(14.8)	
≥70 g/L	130(81.8)	63(78.8)	28(84.8)	23(85.2)	
Platelets					0.547
<150 × 10^9/L	71(44.7)	38(47.5)	13(39.4)	10(37.0)	
≥150 × 10^9/L	88(55.3)	42(52.5)	20(60.6)	17(63.0)	
Neutrophil					0.153
<1.5 × 10^9/L	11(6.9)	7(8.8)	2(6.1)	0(0)	
≥1.5 × 10^9/L	148(93.1)	81(92.0)	31(93.9)	27(100.0)	
Elevated LDH	54(34.0)	28(35.0)	10(30.3)	7(25.9)	0.661
Renal insufficiency^§^	46(28.9)	28(35.0)	8(24.2)	2(7.4)	**0.019**
M protein					0.510
Light chain	21(13.2)	12(15.0)	3(9.1)	4(14.8)	
IgG	97(61.0)	52(65.0)	19(57.6)	16(59.3)	
IgA	37(23.3)	16(20.0)	10(30.3)	7(25.9)	
IgD	4(2.5)	0(0)	1(3.0)	0(0)	
Light chain					0.309
kappa	73(45.9)	39(48.8)	13(39.4)	16(59.3)	
lambda	86(54.1)	41(51.2)	20(60.6)	11(40.7)	
FISH					NA
Standard‐risk	85(81.0)	41(82.0)	22(78.6)	13(86.7)	
High‐risk^†^	20(19.0)	9(18.0)	6(21.4)	2(13.3)	
1q21 amplification	47(44.8)	22(44.0)	12(42.9)	9(60.0)	
Missing	54	30	5	2	
ECOG PS score					0.565
0–1	73(47.4)	38(48.7)	19(57.6)	12(44.4)	
2–4	81(52.6)	40(51.3)	14(42.4)	15(55.6)	
Missing	5	2	0	0	
DS stage					0.563
I	24(15.1)	10(12.5)	6(18.2)	2(7.4)	
II	32(20.1)	16(20.0)	5(15.2)	8(29.6)	
III	103(64.8)	54(67.5)	22(66.7)	17(63.0)	
ISS stage					0.560
I	40(26.0)	18(23.1)	7(21.9)	10(38.5)	
II	40(26.0)	23(29.5)	9(28.1)	5(19.2)	
III	74(48.0)	37(47.4)	16(50.0)	11(42.3)	
Missing	5	2	1	1	
R‐ISS stage					0.628
I	19(14.4)	9(13.0)	3(10.0)	5(25.0)	
II	79(59.8)	42(60.9)	20(66.7)	11(55.0)	
III	34(25.8)	18(26.1)	7(23.3)	4(20.0)	
Missing	27	11	3	7	
Median treatment cycles (range)	6(1–34)	6(1–11)	4(1–9)	8(2–24)	0.000
Response rate					
sCR/CR	35(25.3)	16(21.6)	13(39.4)	4(16.0)	NA
VGPR	29(21.0)	15(20.3)	5(15.2)	7(28.0)	
PR	45(32.6)	23(31.1)	13(39.4)	9(36.0)	
MR	22(15.9)	14(18.9)	2(6.1)	5(20.0)	
SD	7(5.1)	6(8.1)	0(0.0)	0(0.0)	
≥VGPR	64(46.4)	31(41.9)	18(54.5)	11(44.0)	0.472
ORR (≥PR)	109(79.0)	54(73.0)	31(93.9)	20(80.0)	**0.046**
Missing	21	6	0	2	

*Note*: Bold values indicate statistically significant *p* < 0.05.

Abbreviations: CR, complete response; DS, Durie‐Salmon; ECOG PS, Eastern Cooperative Oncology Group performance status; FISH, fluorescent in situ hybridization; ISS, International Staging System; MR, minimal response; ORR, overall response; PR, partial response; R‐ISS, Revised International Staging System; sCR, stringent complete response; SD, stable disease; VGPR, very good partial response.

† High‐risk was defined as the presence of t(4;14) or t(14;16) or 17p deletion.

‡ The *p* values are testing for the significance of differences among groups Bd, Bd + X, and Rd.

§ Renal insufficiency was defined as the serum creatinine of >177 μmol/L.

### 3.2 Treatment characteristics

Among them, 147 (92.5%) elderly NDMM patients had received at least one cycle of induction therapy, and the treatment details were illustrated in Figure 1. Bd and Rd are the backbone regimen for elderly MM, while triplet therapies termed Bd + X like VMP, bortezomib/cyclophosphamide/dexamethasone (BCd), and bortezomib/lenalidomide/dexamethasone (BRd) were prescribed in certain conditions. The frequency of Bd (*n* = 80, 50.3%), Rd (*n* = 27, 17.0%), and Bd + X (*n* = 27, 20.8%) showed different trends depending on the clinical characteristics of the elderly MM patients (Table 1). Bortezomib‐containing regimens were the most commonly prescribed induction therapy for elderly MM in this study. There were no statistically significant differences in clinical characteristics among the three groups, except that patients ≥75 years old seldom received triplet therapy (*p* = 0.001) and only two patients with renal insufficiency received the Rd regimen (*p* = 0.019).

In the Rd group, four (14.8%) patients with hemoglobin less than 70 g/L and 10 (37.0%) patients with platelets less than 150 × 10^9^/L did not satisfy the inclusion criteria for the Rd regimen. However, among the 10 patients, seven had platelets more than 100 × 10^9^/L and the other two patients experienced the withdrawal of Rd treatment due to severe thrombocytopenia. Twenty (74.1%) patients had the ECOG PS score ≤2 and seven (25.9%) presented with an ECOG PS score >2, of whom one patient experienced drug discontinuance due to the complication of pneumonia.

### 3.3 Treatment efficacy

Response assessments were available for 138 elderly MM patients and are summarized in Table 1. In total, 109 (79.0%) and 64 (46.4%) patients achieved the best response of ≥PR and very good partial response (VGPR), respectively. For patients ≥75 years old, 40.6% of the patients achieved VGPR and better and 71.9% achieved PR and better.

The median treatment cycles of Bd, Bd + X, and Rd regimens were six, four, and eight, respectively (*p* = 0.000). The ORR of patients receiving Rd was 80.0%, which was not significantly different from Bd (73.0%, *p* = 0.484) or Bd + X (93.9%, *p* = 0.221). However, the triplet therapy Bd + X showed a superior ORR than Bd (*p* = 0.013). There were no significant differences in the rates of VGPR or better among Bd, Bd + X, and Rd regimens (41.9% vs. 54.5% vs. 44.0%, *p* = 0.472). In the high‐risk elderly MM patients, the rates of ≥VGPR among Bd, Bd + X and Rd regimens were 37.5%, 50.0%, and 47.1%, respectively (*p* = 0.446). For patients with ECOG PS score ≥2 or ≥ 75 years old, there was no statistical significance among Bd, Bd + X, and Rd regimens.

### 3.4 Survival outcomes

The median follow‐up time was 20.8 months for the entire cohort. The median PFS was 24.5 months (95% Confidence interval [CI] 18.2–30.7) and the median OS was not reached, while the estimated 24‐month OS rate was 75.4%. At the time of analysis, 67 (42.1%) patients had progressed, and 34 (21.4%) had died in total.

**FIGURE 3:**
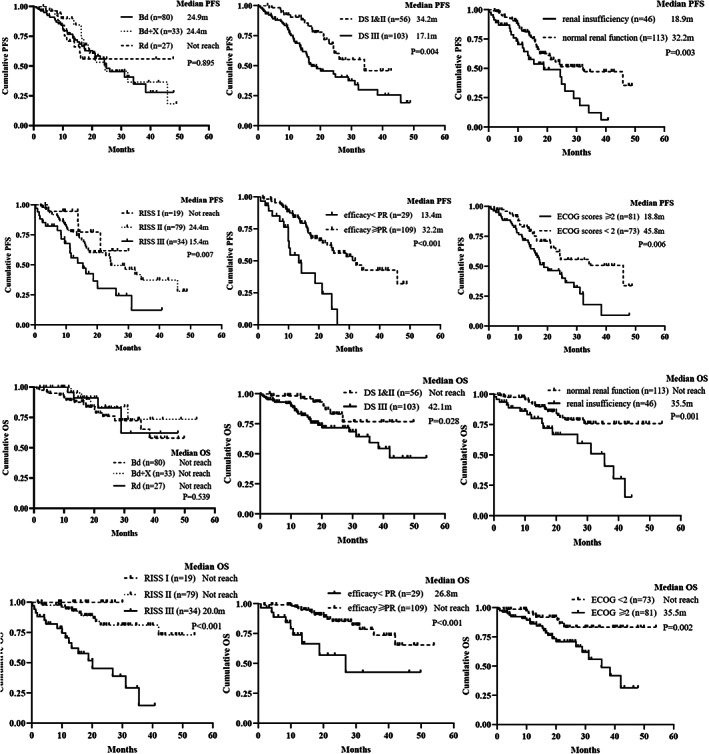
Progression‐free survival and overall survival for the elderly multiple myeloma patients grouped according to different factors.

The median PFS of the Bd, Bd + X, and Rd regimens for the elderly NDMM patients were 24.9 months (95% CI 17.3–32.4), 24.4 months (95% CI 10.8–38.1), and not reached, respectively (*p* = 0.895) (Figure 3). Meanwhile, there were no statistically significant differences in PFS between the Rd and Bd treatment group (*p* = 0.858) or between Rd and Bd + X (*p* = 0.867). The median PFS of all the bortezomib‐containing groups was 24.4 months (95% CI 16.7–32.2) and no statistically significant difference was observed compared with Rd (*p* = 0.947). The median OS in the Bd, Bd + X, and Rd regimens of elderly NDMM were not reached and there were no significant differences in OS among the three groups (*p* = 0.539) (Figure 3). The estimated 24‐month OS rate was 76.0%, 84.0%, and 82.8%, respectively. Similar to the PFS, no statistically significant difference in OS was observed between Bd and Rd groups (*p* = 0.536). Besides, the three regimens showed equivalent PFS and OS no matter in the standard‐risk or high‐risk MM patients, respectively.

The analysis of potential risk factors for PFS and OS in the whole cohort was conducted by the Kaplan–Meier method and Cox proportional hazards model (Figure 3 and Table 2). Univariate analysis identified that platelets (PLT) ≥150 × 10^9/L, Neutrophil ≥1.5 × 10^9/L, ECOG scores ≥2, Renal insufficiency, DS stage III, R‐ISS III, or efficacy ≥PR were potential risk factors for PFS and ECOG scores ≥2, Renal insufficiency, DS stage III, R‐ISS III, or efficacy ≥PR were potential risk factors for OS. Multivariable analysis showed that efficacy ≥PR were independent prognostic factors for PFS, whereas R‐ISS III and efficacy ≥PR were independent prognostic factors for OS.

**TABLE 2:** Univariate and multivariate analyses of risk factors for PFS and OS

Parameters	Univariate analysis for PFS		Multivariate analysis for PFS		Univariate analysis for OS		Multivariate analysis for OS	
	*p* value	HR (95% CI)	*p* value	Adjusted HR (95% CI)	*p* value	HR (95% CI)	*p* value	Adjusted HR (95% CI)
Age ≥ 75 years	0.417	1.25(0.73–2.16)	–	–	0.264	1.53(0.73–3.22)	–	–
Gender (female)	0.663	1.11(0.69–1.81)	–	–	0.494	0.78(0.39–1.58)	–	–
PLT ≥150 × 10^9/L	0.007	0.51(0.31–0.84)	0.123	0.62(0.33–1.14)	0.116	0.58(0.29–1.15)	–	–
Neutrophil ≥1.5 × 10^9/L	0.020	0.39(0.17–0.86)	0.205	0.47(0.15–1.51)	0.157	0.47(0.16–1.34)	–	–
M proteins types	0.186	1.24(0.90–1.71)	–	–	0.395	1.23(0.77–1.97)	–	–
Lights chain types	0.232	1.35(0.83–2.21)	–	–	0.202	1.57(0.79–3.14)	–	–
ECOG scores ≥2	0.007	2.03(1.21–3.39)	0.324	1.39(0.73–2.66)	0.004	3.29(1.47–7.37)	0.263	1.87(0.63–5.57)
DS stage III	0.005	2.20(1.26–3.82)	0.076	1.96(0.93–4.13)	0.034	2.47(1.07–5.68)	0.312	1.88(0.55–6.42)
Renal insufficiency	0.004	2.07(1.26–3.41)	0.230	1.53(0.76–3.08)	0.002	2.96(1.50–5.82)	0.522	1.41(0.49–4.05)
R‐ISS III	0.004	2.26(1.31–3.90)	0.792	1.10(0.54–2.23)	0.000	7.15(3.26–15.70)	**0.003**	5.14(1.75–15.09)
Efficacy ≥PR	0.000	0.29(0.16–0.53)	**0.003**	**0.33(0.16–0.69)**	0.001	0.25(0.11–0.57)	**0.018**	0.28(0.10–0.81)
Rd vs. Bd	0.857	0.94(0.46–1.90)	–	–	0.538	0.71(0.24–2.12)	–	–

*Note*: Bold values indicate statistically significant *p* < 0.05.

Abbreviations: Bd, bortezomib/dexamethasone; ECOG, Eastern Cooperative Oncology Group; OS, overall survival; PFS, progression‐free survival; PR, partial response; Rd, lenalidomide/dexamethasone.

### 3.5 Characteristics of bone marrow regulatory B cells and PET‐CT scan in the elderly NDMM patients

**FIGURE 4:**
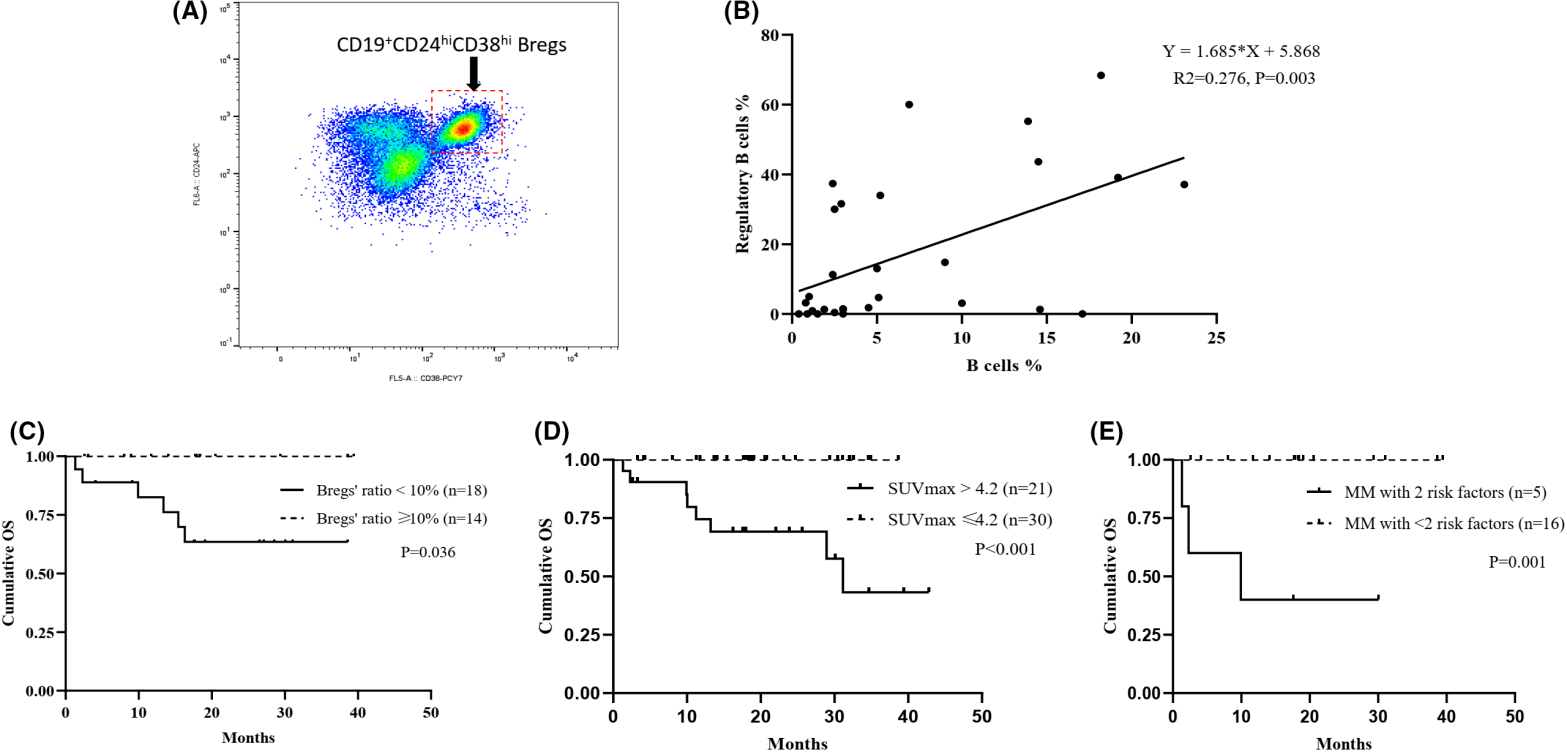
Analysis of bone marrow‐derived regulatory B cells and PET/CT scan in the elderly with multiple myeloma. (A). Bone marrow from MM patients was stained with CD19, CD24, and CD38 by flow cytometry. Bregs are phenotypically identified as a distinct subset of CD19^+^CD24^high^CD38^high^ cells. (B). A statistically significant positive correlation was found between the proportion of B cells and CD19^+^CD24^high^CD38^high^ Bregs in the bone marrow of multiple myeloma. (C). Overall survival curves according to the proportions of regulatory B cells. (D). Overall survival curves according to SUVmax of PET/CT. (E). Overall survival curves based on the number of adverse risk factors (risk factors including Bregs' ratio < 10% and SUVmax>4.2).

Flow cytometry analysis was applied to characterize the BM‐derived CD19^+^CD24^hi^CD38^hi^ Bregs in the elderly MM and data were available in 32 patients (Figure 4A and Table S1), including 15 receiving Bd regimen, two receiving BCd regimen, and six receiving BRd regimen. The median proportions of Bregs within BM CD19^+^B cells were 4.85% (interquartile range 1.00%–33.40%) and the proportion of Bregs was found to significantly positively correlate with the proportion of CD19^+^B cells (*p* = 0.003) (Figure 4B). However, there were no significant differences in Bregs frequencies among the ECOG scores, DS stage, ISS stage, or R‐ISS stage in the elderly NDMM patients, respectively. Furthermore, the ratio of Bregs showed no significant relation to the treatment efficacy (Figure S1). Notably, patients with a Bregs' ratio of <10% showed a significantly worse OS (hazard ratio [HR] 49.7, *p* = 0.036) (Figure 4C) and no significant association with PFS (Figure S1).

Fifty‐seven patients received PET/CT scans at diagnosis and 80.7% presented positive lesions. The median SUVmax was 3.68 (interquartile range 2.85–5.76). The difference in induction efficacy between patients with SUVmax >4.2 or SUVmax ≤4.2 was insignificant. However, the median PFS was shorter in patients with SUVmax >4.2 than in patients with SUVmax ≤4.2 (16.0 m vs. not reached, *p* = 0.136), and SUVmax >4.2 was significantly associated with more inferior OS (HR 112.8, *p* < 0.001) (Figure 4D).

Since Bregs' ratio <10% and SUVmax >4.2 were significantly correlated with more inferior OS, we stratified the elderly MM patients into different groups according to the number of adverse risk factors. Among patients with available Breg and PET/CT scan data, 23.8% had two adverse factors, 33.3% had only one adverse factor, and 42.9% had neither of the two adverse factors. As a result, patients with both Bregs' ratio < 10% and SUVmax >4.2 experienced inferior PFS and OS than the other two groups (*p* = 0.109 and *p* = 0.001, respectively) (Figure 4E). Due to the limited sample size of patients, who underwent both bone marrow Bregs and whole‐body PET‐CT scans, these two variables were not included for multivariable survival analysis in the study.

## 4 DISCUSSION

There is no consensus on the treatment of elderly patients with MM in real‐world practice, although clinical trials have proven the efficacy of novel agents in MM. The management of elderly MM is challenging due to various comorbidities and compromised health status, generally ineligible for clinical trials limiting the evidence for the elderly.^23^ Additionally, in the era of novel agents, no head‐to‐head randomized trials in elderly MM patients have compared clinical outcomes between bortezomib‐containing and lenalidomide‐containing regimens as frontline therapy, which were described by only a few retrospective studies.^24^, ^25^ To provide a valuable reference to stratify the elderly NDMM, we present the ongoing CEMM2021 algorithm in our center, a medical center in Southwest China, which results in comparable short‐term induction efficacy and long‐term survival in patients with higher ECOG PS scores, DS, and ISS stages than patients enrolled in the clinical trials.^26^, ^27^ According to the study, for elderly NDMM, how to enlarge the well‐tolerating candidates on Rd to reduce treatment withdrawal and finally benefit from Rd should be updated, especially compared with bortezomib‐based regimens.

Under the guidance of the CEMM2021 model based on elderly patients' general condition, less than 20% of elderly NDMM are eligible to initiate the continuous Rd regimen as frontline therapy, which was solidly supported by the FIRST trial.^27^, ^29^, ^30^ In a retrospective study of 394 patients over 75 years old with NDMM in the Mayo Clinic, Rd was the most commonly used regimen, which was different from our research, but the PFS and OS were statistically significantly different in different treatment groups.^31^ Recently, a pooled analysis of two clinical trials for the transplant‐ineligible elderly NDMM in which Rd induction followed by lenalidomide maintenance^32^ or VMP as primary therapies^33^ showed that patients over 75 years of age might benefit more from Rd induction followed R maintenance.^34^ However, not all the elderly MM patients were suitable for the Rd regimen in real‐world practice since the eligibility criteria of clinical trials exclude patients with frailty, severe anemia, renal insufficiency, and comorbidities. One report on real‐world outcomes of 1156 transplant‐ineligible NDMM patients with the median age of 72.3 years old from the Canadian Myeloma Research Group database showed that only 18% received Rd and 82% received bortezomib‐containing regimens while outcomes with the BD/P were the most inferior.^25^ Similarly, in our study, 20% of the elderly MM had ECOG score ≥3 and twice as many patients had elevated LDH, while the ECOG score was almost no more than three, and patients with ISS III and elevated LDH were 40% and 16%, respectively, in the FIRST trial,^27^ which may explain the reason for the low rate of Rd regimen as frontline therapy in our study.

On the other hand, by bortezomib‐based primary treatment, the majority of elderly NDMM in the study, not satisfying all the strict criteria mentioned above to initiate Rd, achieved comparable response and survival benefits. Similarly, a real‐world study in transplant‐ineligible NDMM from Spain revealed that bortezomib‐containing regimens (81.7%) were the most commonly prescribed primary therapy, and only 3.6% of patients received the Rd regimen.^25^ Meanwhile, the baseline characteristics of the patients were similar to our study, and among the patients with a mean age of 75.6 years, 47.4% had ECOG score 2–4 and 25.1% had high‐risk cytogenetic abnormalities. The median PFS and OS were 15.3 m and 33.5 m, respectively.^25^ Besides, a retrospective study based on the Surveillance, Epidemiology and End Results (SEER) evaluated the year‐to‐year changes in a cohort of MM patients age 66 or older between 2007 and 2011, which found that the usage of proteasome inhibitors increased significantly and replaced immunomodulatory drug‐based regimens.^34^ According to these studies, proteasome inhibitors still play an essential role in the induction therapy of elderly MM.

It is also noteworthy that the management of older patients with MM, presenting a highly heterogeneous nature, is often challenging due to insufficient recruitment for clinical trials. And data in real‐world settings are not yet sufficient to meet the demand, specifically, merely based on only a few reports that enrolled several hundreds of elderly NDMM patients among the globally dispersing medical centers and one Canadian study enrolled 1156 transplant‐ineligible MM from the national web‐based database.^25^ In this study, the CEMM2021 algorithm for elderly NDMM in Southwest China resulted in comparable induction efficacy and survival benefits among these universally available regimens. Besides, we incorporated Bregs into the prognostic system for MM.

This study also confirmed that for elderly patients with MM, the proportion of BM‐derived Bregs is related to the OS, while the proportions of Bregs and SUVmax were significantly associated with OS but not PFS, which was similar to our previous studies.^19^ It may indicate that Bregs may negatively affect survival independent of tumor progression, and OS was valued as a more critical cancer trial outcome compared with PFS.^35^ Bregs, a population of suppressor B cells, were initially described in autoimmune disease and much progress has been made to characterize their phenotype and function in the tumor microenvironment over the past decade.^14^, ^36^ Bregs can negatively regulate immune response by producing a series of suppressive cytokines, affecting cancer cell growth.^9^, ^13^ Bregs are also crucial in maintaining immune tolerance and suppressing inflammation in the BM microenvironment.^37^ However, the characteristics of Bregs in MM are poorly understood. The continuous work on examinations of Bregs for MM is in process at our center, ensuring successive emergence of the related findings. Our previous studies have found that in MM, Bregs of the phenotype as CD19^+^CD24^hi^CD38^hi^ are distinguished from other cells in BM but not in peripheral blood, conferring an immunosuppressive microenvironment via IL‐10 secretion.^15^ Meanwhile, patients with monoclonal gammopathy of undetermined significance showed significantly increased proportions of Bregs than MM.^15^ Bregs' ratios were also found to be positively correlated with the preserved B cells in MM, while the higher percentages of Bregs may be associated with better prognosis.^19^ Differing from our previous reports on the whole NDMM population *taking no account of age and transplantation implementation, the study further focuses on the elder subpopulation of NDMM, which is probably* ineligible for transplantation. Additionally, there are great variabilities among the enrollment time, follow‐up time, and both inclusion and exclusion criteria, showing minimal overlap across the research databases. Similar conclusions have been reproducibly reached, demonstrating that the prognostic role of Bregs is reliable in NDMM. There still exist limitations in our CEMM 2021 treatment model, such as the limited sample size, so more cases from other centers should be validated. In addition, the observational study on targeted therapies releases a quotable real‐world estimation of compromised survival for Asian elder patients with MM, while contemporaneously the relevant clinical trials on immune therapies display an obvious survival improvement. There is presently an urgent need to upgrade by shifting toward immune‐based development, thereby affecting the sustained enrollment into the CEMM 2021 treatment model. The ongoing studies, including a retrospective multicenter evaluation and prospective cohort analyses, will contribute to a general reassessment of the corresponding cases.

The CEMM 2021 treatment model provides a solid basis for small molecular inhibitors for elderly cases with MM and requires an urgent upgrade to improve survival for currently diagnosed patients in the near future. To be specific, the treatment of MM has entered the era of cellular immunotherapy and antibody‐containing immunotherapies have been recently recommended as the frontline therapy for the elderly MM by the guidelines.^23^, ^38^, ^39^ Meanwhile, particular attention would be consistently paid to the potential prognostic significance of immune‐related factors, including Bregs during the transitional era for transplant‐ineligible NDMM.^40^, ^41^

## 5 CONCLUSION

With the aging of the population, the interest in studies concerning elderly patients with NDMM, the most common malignant plasma cell disorder, has dramatically increased. Our data reported in Southwest China herein indicates that the treatment algorithm described above might provide a valuable reference for the guidance of therapeutic strategies in elderly NDMM. Besides, the combination of the model with Bregs led to practical and clinically meaningful stratification in the contemporary treatment for the elder NDMM.

## AUTHOR CONTRIBUTIONS

**Wenjiao Tang:** Data curation (equal); investigation (lead); writing – original draft (lead); writing – review and editing (equal). **Yan Li:** Data curation (equal); resources (equal). **Zhongqing Zou:** Data curation (equal); methodology (equal); resources (equal). **Jian Cui:** Data curation (equal); methodology (equal); resources (equal). **Fangfang Wang:** Methodology (equal); resources (equal). **Yuhuan Zheng:** Resources (equal); supervision (equal). **Li Hou:** Resources (equal). **Ling Pan:** Resources (equal). **Bing Xiang:** Resources (equal). **Hong Chang:** Resources (equal). **Li Zhang:** Conceptualization (lead); funding acquisition (equal); project administration (lead); resources (equal); supervision (equal); writing – review and editing (equal). **Ting Niu:** Conceptualization (supporting); resources (equal); supervision (equal); writing – review and editing (supporting).

## FUNDING INFORMATION

This study was supported by the National Natural Science Foundation of China for the general program (817780218) and the Natural Science Foundation of Sichuan Province (2022NSFSC1299).

## CONFLICT OF INTEREST

There is no conflict of interest to declare.

## ETHICS APPROVAL

This study was approved by the ethics committee of West China Hospital, Sichuan University (WCHSCU) and all the procedures involving human participants have complied with the Helsinki Declaration.

## CLINICAL TRIAL REGISTRATION NUMBER

chiCTR2000029925.

## INFORMED CONSENT

All the participants provided written informed consent.

## Supporting information


Figure S1

Table S1

## Data Availability

The data that support the findings of this study are available on request from the corresponding author.
