# Supplementary figures and images for: A stratified therapeutic model incorporated with studies on regulatory B cells for elderly patients with newly diagnosed multiple myeloma
Source: Cancer Med. 2022 Sep 20;12(3):3054–67. doi: 10.1002/cam4.5228 (PMC9939179; doi:10.1002/cam4.5228)

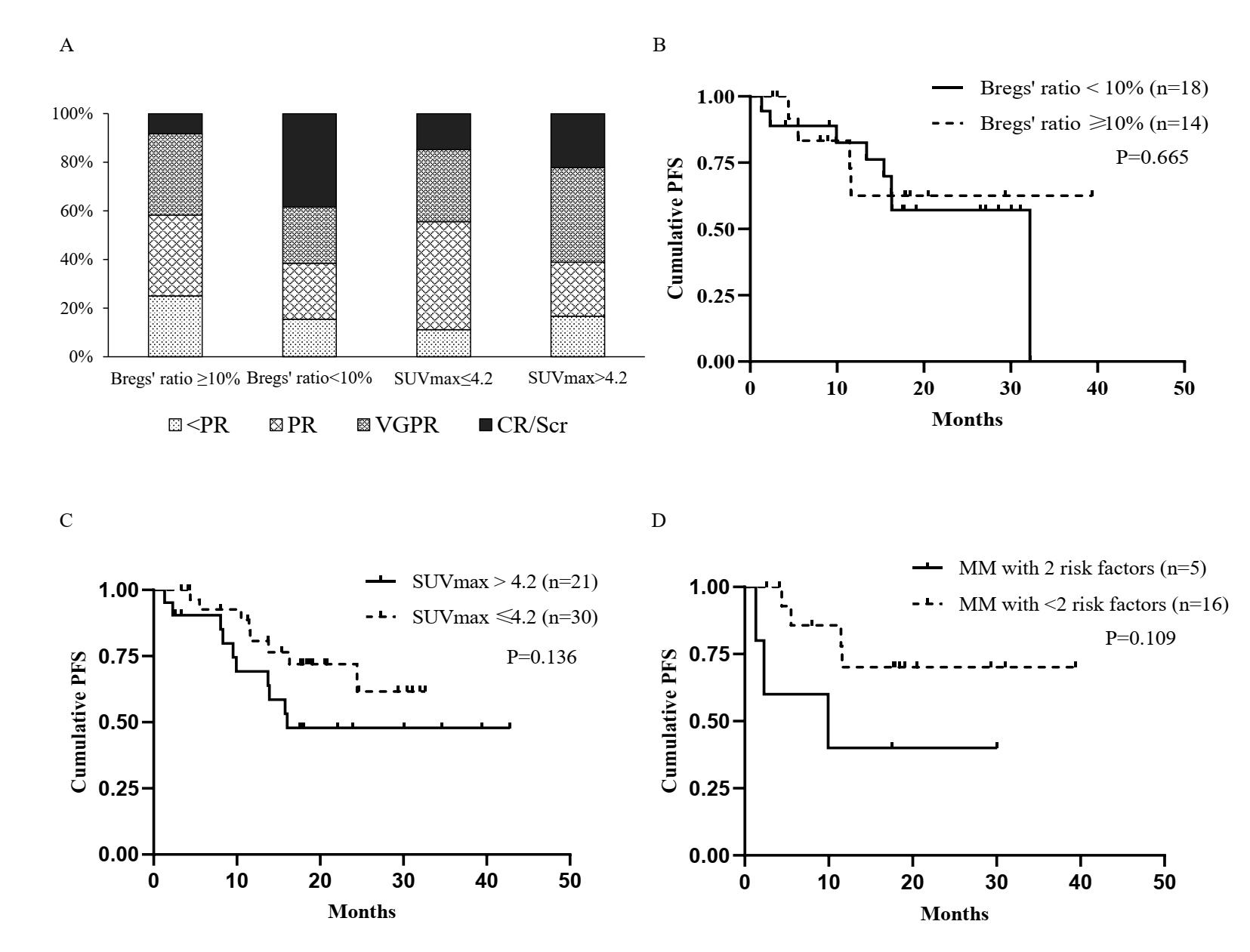

Supplement: Supplementary file 1 — Figure S1 [file CAM4-12-3054-s001.jpeg]
