# Supplementary material for: A stratified therapeutic model incorporated with studies on regulatory B cells for elderly patients with newly diagnosed multiple myeloma
Source: Cancer Med. 2022 Sep 20;12(3):3054–67. doi: 10.1002/cam4.5228 (PMC9939179; doi:10.1002/cam4.5228)
Supplement: Supplementary file 2 — Table S1 [file CAM4-12-3054-s002.docx]

SuppTable 1. Characteristics of regulatory B cells of the elderly multiple myeloma patients

| **Characteristics– no (%)** | **Patients with Bregs ＜10%**  **(n=18)** | **Patients with Bregs ≥10%**  **（n=14）** | **P value** |
| --- | --- | --- | --- |
| Median age (range) | 68(65-77) | 68(65-78) | 0.615 |
| Gender (Male/Female) | 13/5 | 8/6 | 0.465 |
| Elevated LDH | 5(27.8) | 3(21.4) | 0.681 |
| Renal insufficiency | 4(22.2) | 3(21.4) | 0.957 |
| M protein  light chain  IgG  IgA  IgD | 0(0)  13(72.2)  3(16.7)  2(11.1) | 3(21.4)  8(57.1)  3(21.4)  0(0) | 0.123 |
| Light chain  kappa  lambda | 6(33.3)  12(66.7) | 7(50.0)  7(50.0) | 0.341 |
| FISH  Standard-risk  High-risk †  missing | 12(75.0)  4(25.0)  2 | 10(83.3)  2(16.7)  2 | 0.595 |
| ECOG PS score  0-1  2-4  Missing | 5(33.3)  10(66.7)  3 | 6(42.9)  8(57.1)  0 | 0.597 |
| DS stage  I  II  III | 1(5.6)  1(5.6)  16(88.9) | 2(14.3)  3(21.4)  9(64.3) | 0.242 |
| ISS stage  I  II  III  Missing | 5(29.4)  4(23.5)  8(47.1)  1 | 3(21.4)  6(42.9)  5(35.7)  0 | 0.518 |
| R-ISS stage  I  II  III  Missing | 2(12.5)  11(68.8)  3(18.8)  2 | 2(16.7)  7(58.3)  3(25.0)  2 | 0.850 |
| Response Rate  ≥VGPR  ORR (≥PR)  Missing | 8(61.5)  11(84.6)  5 | 5(41.7)  9(75.0)  2 | 0.320  0.548 |

† High-risk was defined as the presence of t(4;14) or t(14;16) or 17p deletion.

FISH: fluorescent in situ hybridization; ECOG PS: Eastern Cooperative Oncology Group performance status, DS: Durie-Salmon; ISS: International Staging System; R-ISS: Revised International Staging System; sCR: stringent complete response; CR: complete response; VGPR: very good partial response; PR: partial response; MR: minimal response; SD: stable disease; ORR: overall response
